# Anterior cervical transpedicular screw fixation system in subaxial cervical spine: A finite element comparative study

**DOI:** 10.1097/MD.0000000000029316

**Published:** 2022-07-22

**Authors:** Jie Li, Kaifeng Gan, Binhui Chen, Yilei Chen, Jinjiong Hong, Dikai Bei, Tengdi Fan, Minzhe Zheng, Liujun Zhao, Fengdong Zhao

**Affiliations:** a Department of Orthopedics surgery, Ningbo Medical Center Li Huili Hospital, Affiliated to Ningbo University, Ningbo 315040, Zhejiang, China; b Department of Orthopedics, Sir Run Run Shaw Hospital, School of Medicine, Zhejiang University, Hangzhou 310016, Zhejiang, China; c Department of spinal surgery, Ningbo 6th hospital, Ningbo 315040, zhejiang, China.

**Keywords:** anterior cervical transpedicular screw, subaxial cervical spine, finite element study

## Abstract

Multilevel cervical corpectomy has raised the concern among surgeons that reconstruction with the anterior cervical screw plate system (ACSPS) alone may fail eventually. As an alternative, the anterior cervical transpedicular screw (ACTPS) has been adopted in clinical practice. We used the finite element analysis to investigate whether ACTPS is a more reasonable choice, in comparison with ACSPS, after a 2-level corpectomy in the subaxial cervical spine. These 2 types of implantation models with the applied 75 N axial pressure and 1 N • m pure moment of the couple were evaluated. Compared with the intact model, the range of motion (ROM) at the operative segments (C4–C7) decreased by 97.5% in flexion-extension, 91.3% in axial rotation, and 99.3% in lateral bending in the ACTPS model, whereas it decreased by 95.1%, 73.4%, 96.9% in the ACSPS model respectively. The ROM at the adjacent segment (C3/4) in the ACTPS model decreased in all motions, while that of the ACSPS model increased in axial rotation and flexion-extension compared with the intact model. Compared to the ACSPS model, whose stress concentrated on the interface between the screws and the titanium plate, the stress of the ACTPS model was well-distributed. There was also a significant difference between the maximum stress value of the 2 models. ACTPS and ACSPS are biomechanically favorable. The stability in reducing ROM of ACTPS may be better and the risk of failure for internal fixator is relatively low compared with ACSPS fixation except for under lateral bending in reconstruction the stability of the subaxial cervical spine after 2-level corpectomy.

## 1. Introduction

Robinson et al reported Anterior Cervical Discectomy and Fusion (ACDF) as a therapy for single-level cervical disc disease for the first time in 1958.^[1]^ In most traumatic, degenerative, and pathologic disorders of the cervical spine, the compression frequently lies anteriorly and the anterior approach is commonly referred to as resection of these disorders with the use of ACDF.^[2]^ Because of its demonstrated efficacy, in the following decades, ACDF becomes the standard surgery and became prevalent in clinical practice. Moreover, the fast development of ACDF has resulted in the application of this surgical technique in multilevel cervical disc disease.^[3]^ At the same time, the challenge that surgeons face is that as the operative segments increase, especially in the case of multilevel Anterior Cervical Corpectomy and Fusion (ACCF), the stability of cervical spine reconstruction with current anterior cervical spine screw-plate systems is limited.^[4]^ It is reported that multilevel corpectomy without posterior fixation accounts for a high incidence of failure.^[5,6]^ In this situation, to enhance the stability, additional posterior transpedicular or lateral mass fixation is required even though a second surgery can increase potential surgical risk.^[7]^ As an alternative, some scholars have proposed that the posterior fixation may be avoided as long as the primary construct stability of anterior instrumentation increases, for instance, through increased construct rigidity with anterior-only instrumentations via anterior transpedicular screw-and-plate (ATPS) that anchor screw to pedicle by anterior approach.

**Figure 1.**
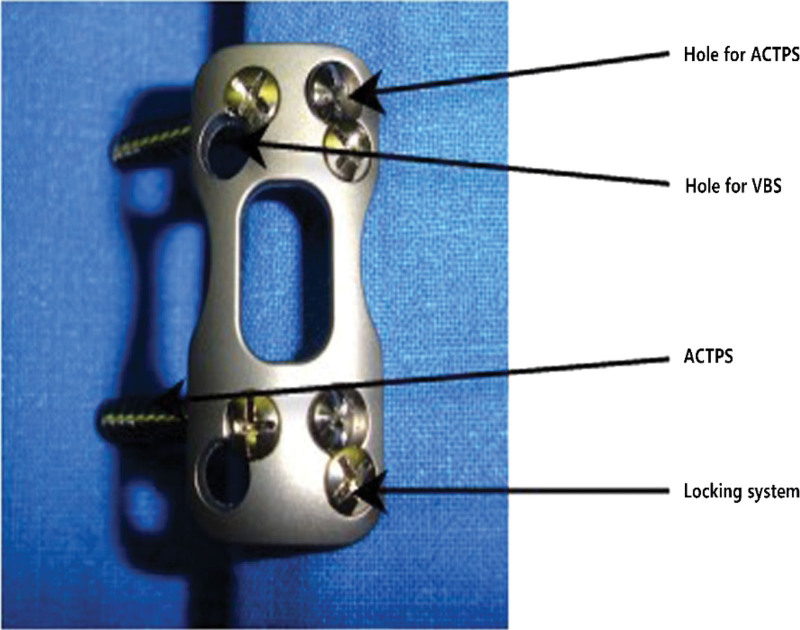
Patented anterior cervical pedicle screw plate system.

In 2008, Koller et al first suggested the new concept - anterior cervical transpedicular screw (ACTPS) and confirmed its feasibility in the subaxial cervical spine through anatomical study.^[8]^ Then they conducted a series of studies to prove that the pull-out strength ACTPS is 2.5 times more than that of the normal anterior vertebral screws (VBS).^[9]^ Since then, some surgeons have applied ACTPS to reconstruct the subaxial cervical spine and achieved reliable clinical outcomes.^[10–13]^ At present, however, there is no dedicated ACTPS system. Surgeons normally use unilateral ACTPS or ACTPS plus AXIS plate fixation instead, though neither of them meets the requirements of pedicle screw placement in the ACTPS system. To this end, we have designed a new type of ACTPS system (ZL 201110357211.7) suitable for the subaxial cervical spine (Fig. 1).^[14]^ This patented system has a locking device and accepts ACTPS, which can be placed at a certain tilted angle, on 1 side and VBS on the other side. In this study, we compared biomechanical features about the range of motion (ROM), bone graft stresses, and bone-screw stresses of our patent ACTPS with the traditional anterior cervical screw plate system (ACSPS) in the subaxial cervical spine after 2-level corpectomy using the finite element analysis. We hypothesize that ACTPS offers an alternative method for treating multilevel cervical disc disease.

## 2. Material and Method

### 2.1. Establishment of the intact model

With the signed consent form and the approval of the Ethics Committee, a 28-year-old healthy male volunteer (175 cm height, 64 kg weight) received X-ray films to exclude conditions such as cervical spine deformities, fractures and unstable situations. 64-slice spiral CT was used to collect the image data from C1 to T1 in the volunteer, which were saved in DICOM format. The raw CT data were transferred into a computer, of which C3–C7, the subaxial cervical spine, were transformed to STL format by Mimics 10.0 software. Then we used Rapidform XOR3 software to repair, denoise and pave the images before converting them to a surface model, which was then processed by CATIA 5V19 software to obtain the final 3-dimensional model of the subaxial cervical spine.

**Figure 2.**
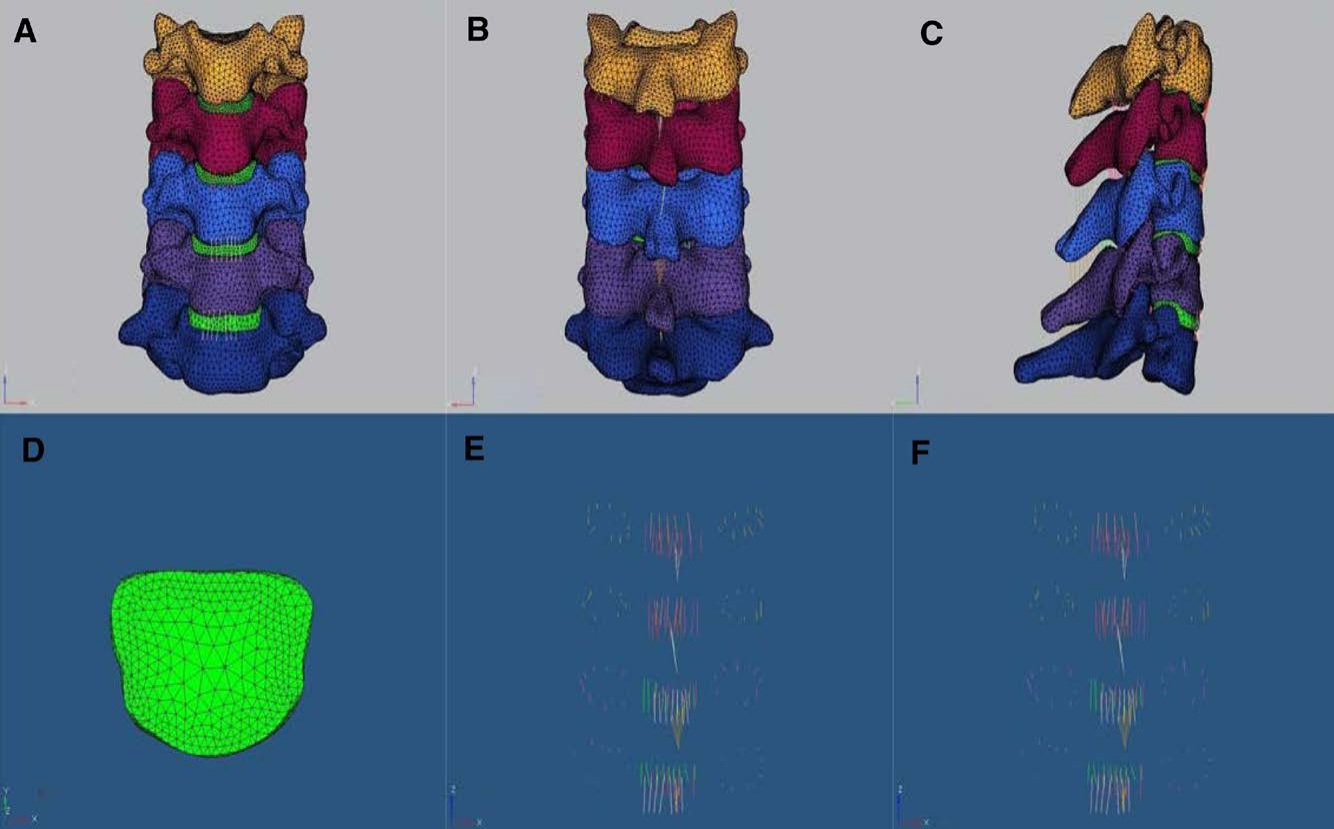
The intact model. (A) Front view of the intact model before being meshed. (B–E): Front view, lateral view, coronal view and rear view of the intact model after being meshed, respectively.

**Figure 3.**
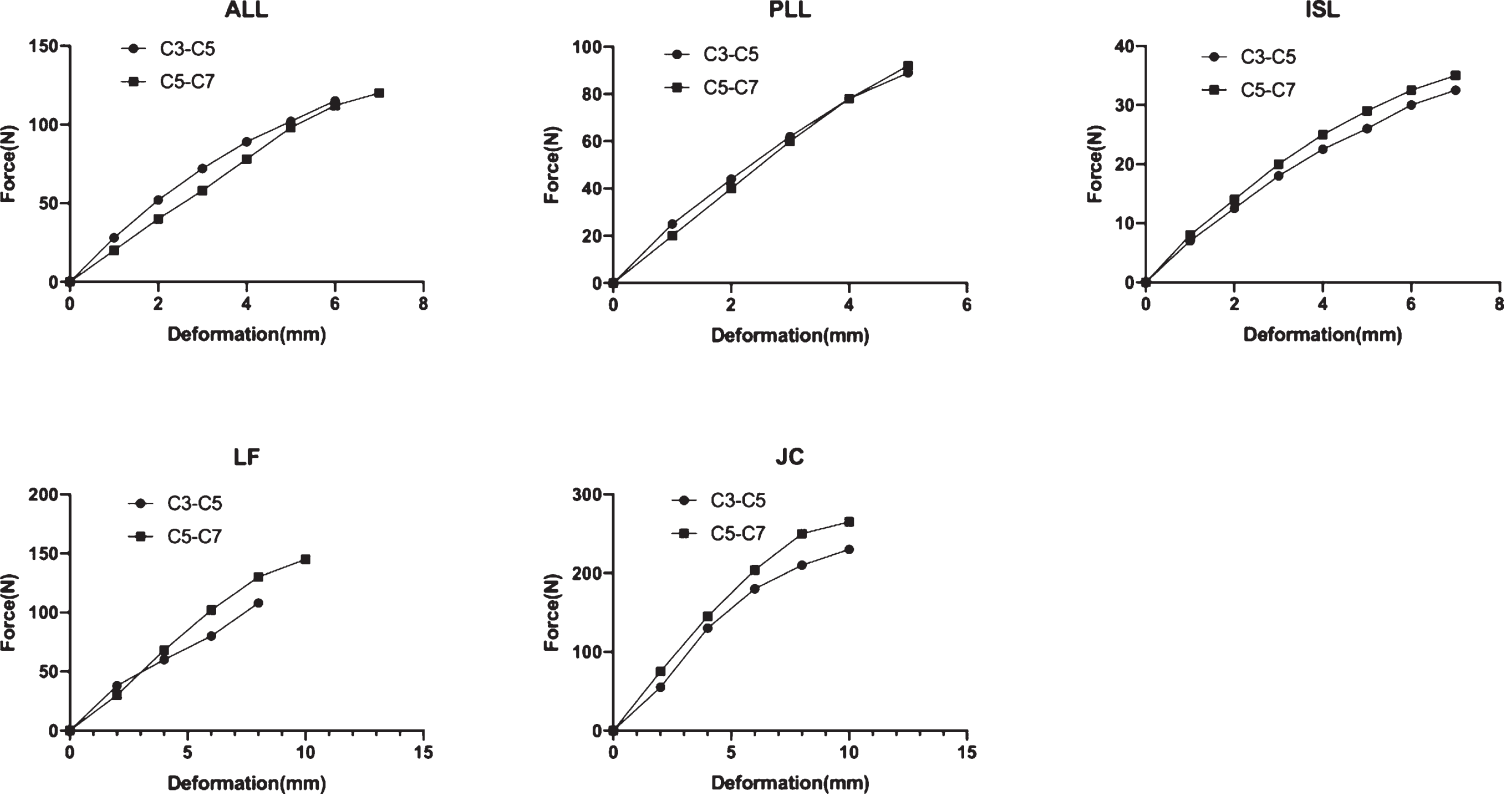
Ligament properties for the nonlinear elastic curve, values of deformation (mm) vs equivalent force (N) for the cervical spinal ligaments. ALL = anterior longitudinal ligament, ISL = interspinal ligament, JC = Joint capsular ligament, LF = ligamentum flavum, PLL = posterior longitudinal ligament.

The intact model was meshed by Hypermesh10.0 software with a Jacobian ratio above 0.6. More specifically, cortical bone, cancellous bone and endplates adopted the tetrahedron elements (C3D4) unit, all the ligaments adopted combined 39 units, the annulus adopted the Rebar unit and the nucleus adopted the fluid unit. Ligaments including anterior longitudinal ligament, posterior longitudinal ligament, capsular ligament, interspinous ligament, supraspinous ligament was established in the intact model (Fig. 2). All the ligaments were modeled as tension-only connectors, and their attachments, courses and mechanical characteristics were determined from the previous literature.^[15–18]^ Material properties are listed in Table 1 and Figure 3.^[19–22]^

**Table 1:** Material Properties of the Human Cervical Spine in the Finite Element Models.^[19–22]^

Structure	Young modulus (MPa)	Poisson ratio
Cortical bone	12,000	0.30
Cancellous bone	100	0.20
Posterior elements	600	0.30
Annulus	4.7	0.45
Nucleus	1666.7*	
Collagenous fibers	500	0.30
Ligaments	Nonlinear elastic curve†	
Titanium implantations	122,000	0.34

* Volume modulus (MPa).

† Please refer to Fig. 3 for details.

### 2.2. Validation of the intact model

**Figure 4.**
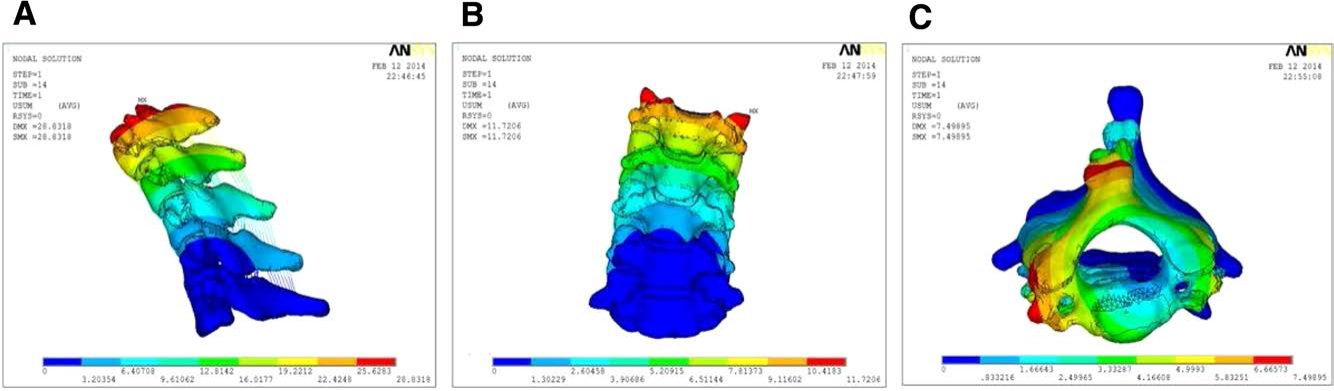
Moment loads of 1.0 N·m was applied over nodes on the C3 superior surface. Following load of 75 N was loaded to the superior surface of the C3, which imitates the weight of the head. (A) Calibration of flexion–extension, (B) calibration of lateral bend- ing, (C) calibration of axial rotation.

The model was imported to the design modeler (DM) module of ANSYS V14.0 software. The C7 inferior endplate was held still in all directions, while C3 could move freely. The method introduced by Panjabi et al was used to determine the vertebral movement angle.^[23]^ Moment loads of 1.0 N·m were applied to the superior surface of the C3. Then a load of 75 N was loaded to the superior surface of the C3, which simulate the weight of the head, so that it could flex, extend, bend laterally and rotate as it would under the physiological state (Fig. 4).^[24]^ Validation was verified by measuring the range of motion (ROM) of each vertebra and comparing them with the in vitro biomechanical test and previous finite element analysis results.^[25–27]^ Moreover, the von Mises stress program was used to display the stress distribution of the intact model during the respective movements, the color from blue to red indicated the stress intensity by degrees and the different color range indicated the respective stress area below Von Mises stress program.

### 2.3. Establishment of the implantation models

**Figure 5.**
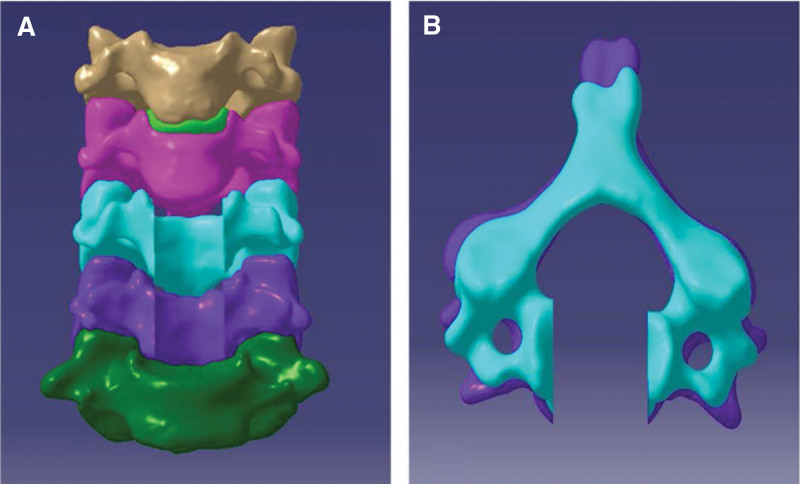
From anterior view (A) and superior view (B) of intact model after C5, C6 corpectomy.

**Figure 6.**
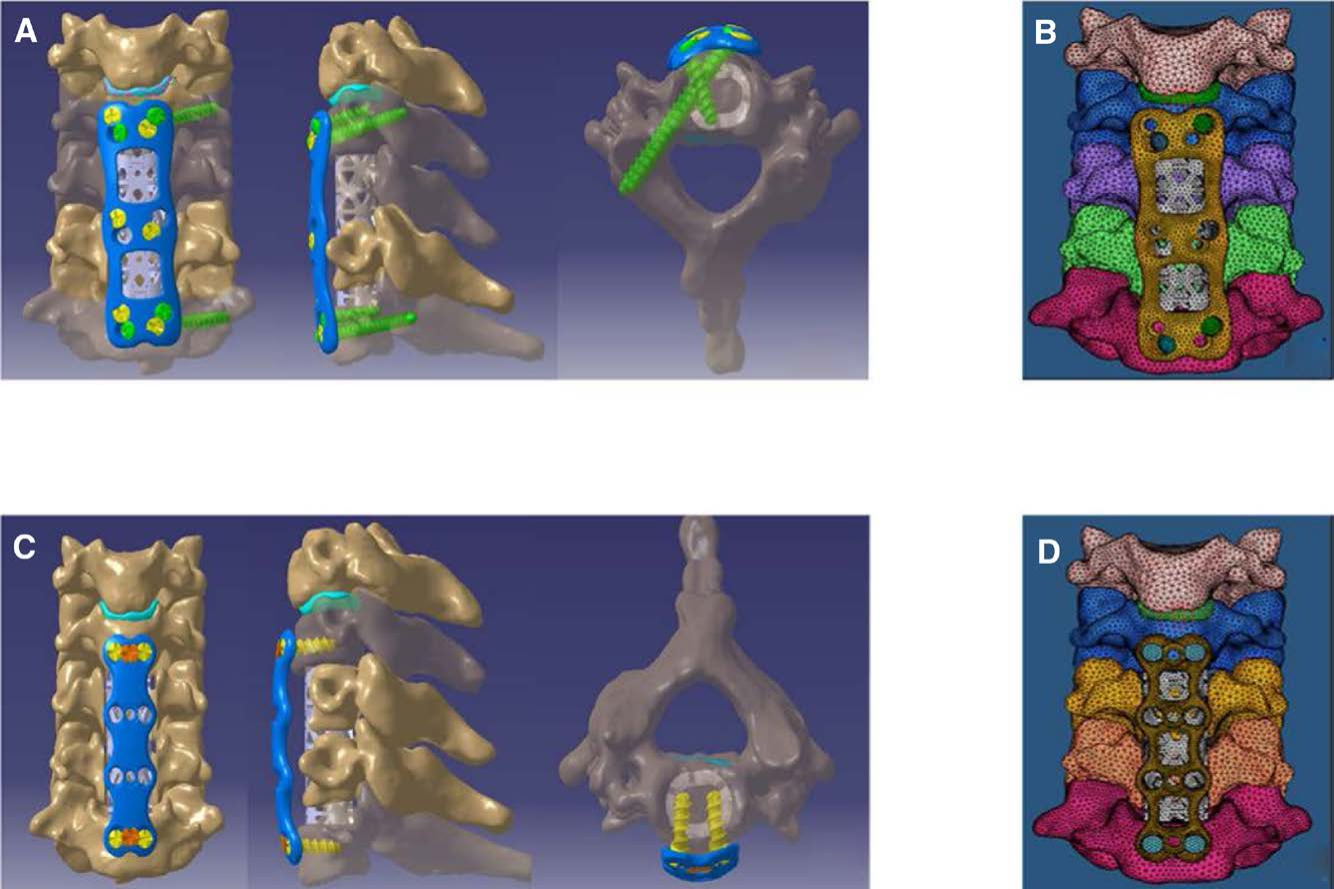
The implantation models. (A–B) Anterior, lateral and superior views of the ACTPS model, (C–D) Anterior, lateral and superior views of the ACSPS model.

To simulate the surgical modalities, the following were excised: anterior longitudinal ligament, C4/5, C5/6 and C6/7 discs, part of the C5 and C6 vertebrae as well as posterior longitudinal ligament (Fig. 5). The width of the decompression slot was 15 mm and we preserved the C4 lower endplate and the C7 upper endplate.^[28]^ CATIA 5V19 software was used to establish the ACTPS model and the ACSPS model with titanium mesh. The dimension (diameter × Length) of VBS was 3.5 mm and 16 mm, while that of ACTPS was 3.5 mm and 30 mm. ACTPS were implanted into the corresponding vertebrae at the same angle as was reported by Xu et al,^[10]^ which is 15° both in the cross-sectional plane and sagittal plane (Fig. 6A, B). Mechanical properties of titanium internal fixation devices are showed in Table 1. Then the models were meshed into C3D4, followed by adding accessory structures according to the intact model. As a result, the total number of nodes and elements in the ACTPS model was 43,802 and 159,548 respectively, while it was 41,455 and 148,514 respectively in the ACSPS model (Fig. 6C, D).

### 2.4. Three-dimensional finite element analysis of the implantation models

The models have also been imported into ANSYS 14.0 software and examined under the aforementioned conditions in the intact model. The maximum stress value, ROM at the operative segment (C4–C7) and the adjacent segment (C3/C4) as well as Von Mises stress program was recorded.

## 3. Results

### 3.1 . Validation of the intact model

**Figure 7.**
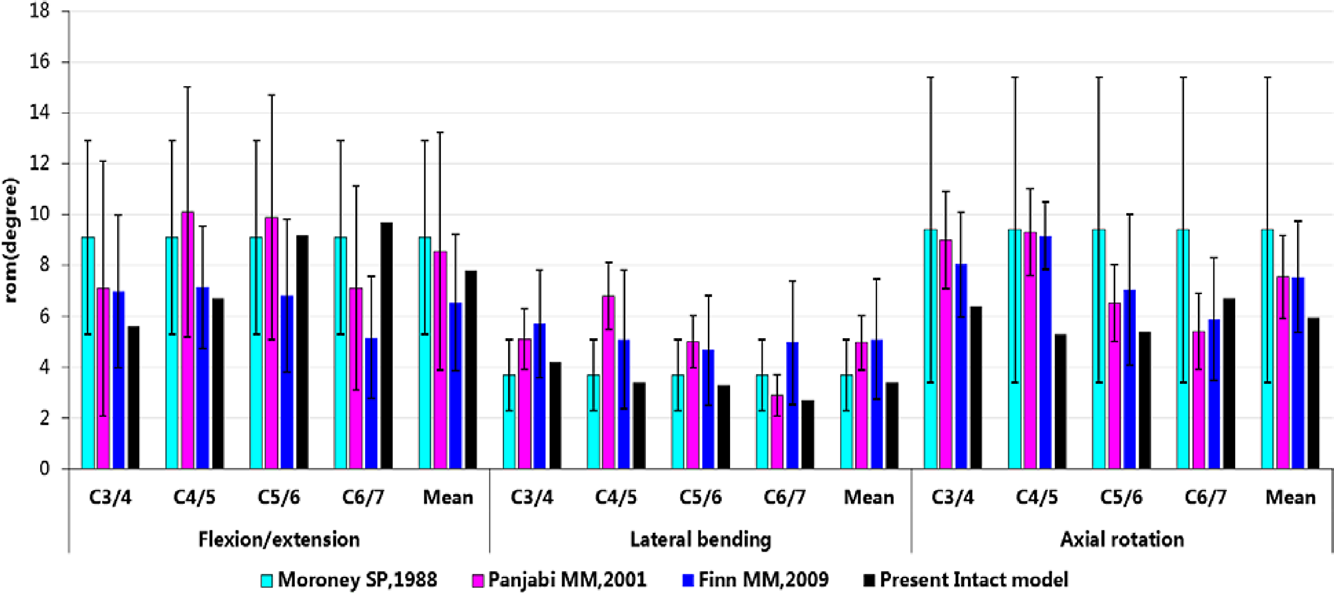
ROM of present intact cervical spine and published in vitro experimental data to validate intact cervical segment on the basis of kinematic similarity. ROM indicates range of motion.

The intact model established in this experiment, including 23,612 nodes and 85,832 elements, trends similar to those previously reported. The ranges of ROM are generally consistent with experimental studies by Moroney et al, Panjabi et al and the FE studies by Finn et al.^[25–27]^ Detailed comparison as was shown in Figure 7, suggesting that this model has good accuracy and can be used for finite element analysis.

### 3.2. Changes in ROM on the ACTPS and ACSPS fixed segments

**Figure 8.**
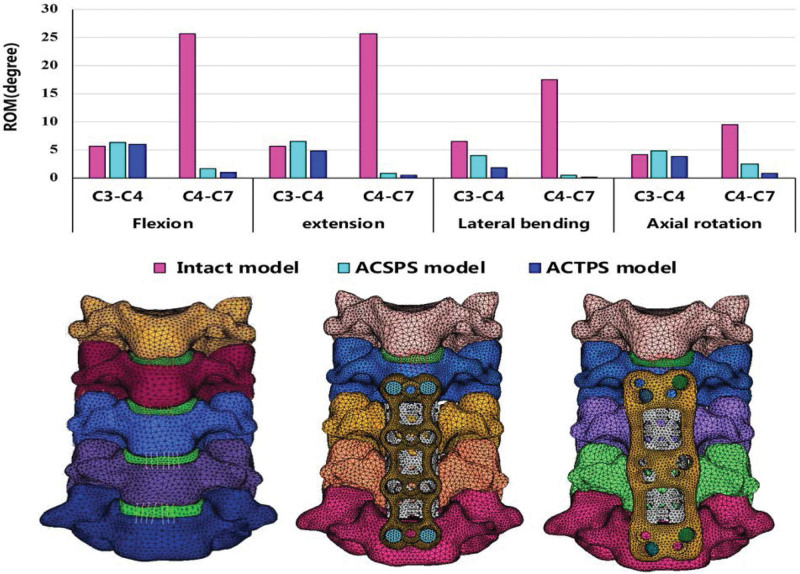
ROM in flexion, extension, lateral bending and rotation in intact model, ACSPS model and ACTPS model groups.

When 75 N axial pressure and 1 N • m pure moment of the couple was applied at the surface of the C3 superior endplate to imitate the movement of cervical spine flexion, extension, axial rotation, and lateral bending, the ROM at C4–C7 in the ACTPS model was smaller than that in both the ACSPS model and the intact model in all motions (Fig. 8). Compared with the intact model, the ROM at C4–C7 decreased by 97.5% in flexion-extension, 91.3% in axial rotation, and 99.3% in lateral bending in the ACTPS model, whereas it decreased by 95.1%, 73.4%, 96.9% in the ACSPS model respectively. ACTPS showed better biomechanical stability compared with ACSPS.

### 3.3. Effect of ACTPS and ACSPS fixation on cephalad intervertebral ROM

Both ACTPS and ACSPS affect the ROM of the adjacent cephalad intervertebral disc. The present experiment shows that the ROM at C3/4, which is the cephalad disc adjacent to the fixed segment, in the ACTPS model was decreased by 3.3% in flexion-extension, 10.2% in axial rotation, and 71.3% in lateral bending compared with the intact model. At the same time, the ROM at C3/4 was decreased by 37.5% in lateral bending, but was increased by 14.3% and 15.2% during flexion-extension and axial rotation, respectively, in the ACSPS model. ACTPS has the potential benefits of reducing cephalad adjacent disc degeneration compared to ACSPS.

### 3.4. Von mises stress nephogram

**Figure 9.**
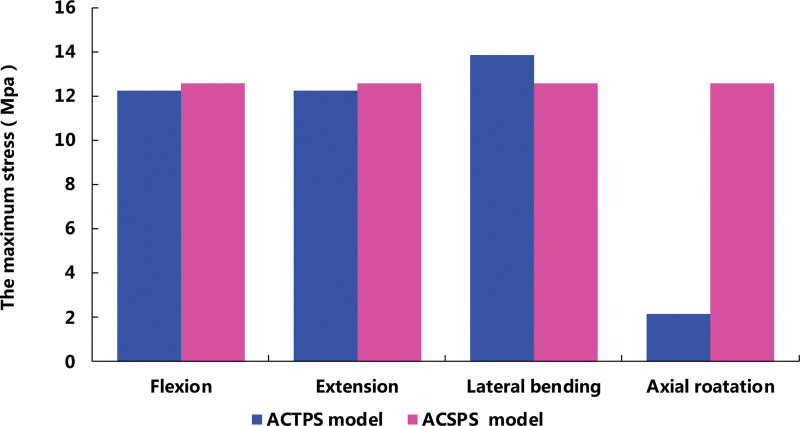
The maximum stress value of fixators in the ASPS and AVBS models.

**Figure 10.**
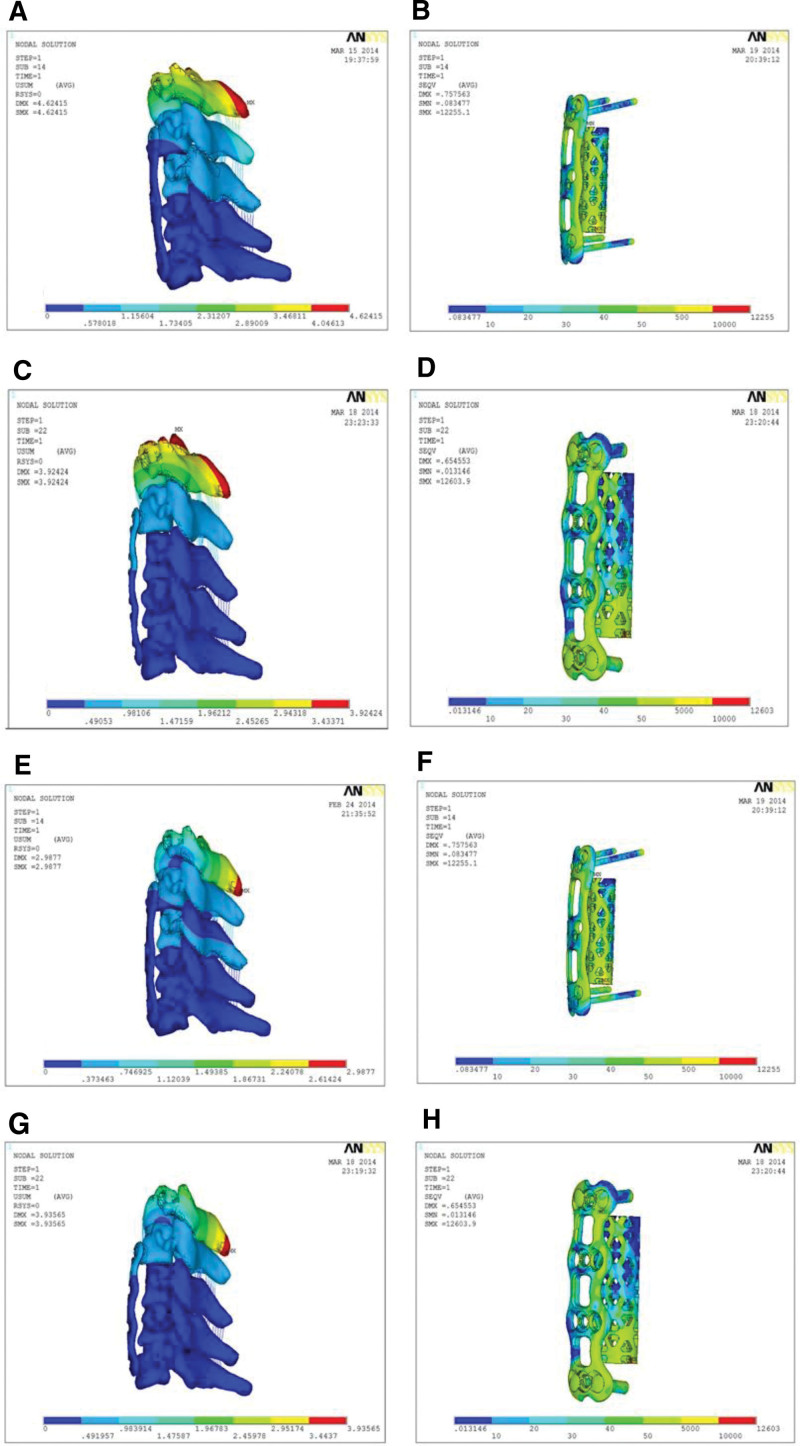
Stress contour map of ACTPS model and ACSPS model under conditions of axial loading: (A,B) stress distribution in the ACTPS model under flexion, (C,D) stress distribution in the ACSPS model under flexion, (E,F) stress distribution in the ACTPS model under extension, (G,H) stress distribution in the ACSPS model under extension, (I,J)stress distribution in the ACTPS model under lateral bending, (K,L) stress distribution in the ACTPS model under lateral bending, (M,N) stress distribution in the ACTPS model under axial rotation, (O,P) stress distribution in the ACSPS model under axial rotation. The ACTPS model showed relatively even stress distribution. The ACSPS model showed stress concentration at the interface between the screws and the titanium plate.

The maximum stress value in the ACTPS model was smaller compared with the ACSPS model under flexion-extension and axial rotation, except for lateral bending (Fig. 9). Von Mises stress program depicted the load-and-displacement of the 2 implantation models. The stress distribution in the ACTPS model group seemed to be relatively even, while the stress concentrated on the interface between the screws and the titanium plate in the ACSPS model (Fig. 10). The von Mises stress distributions show a stress concentration in the contact area between the titanium mesh and the upper endplate of the inferior vertebral body during motion in the lateral flexion and rotation directions in the ACTPS group (Fig. 10J, N). These results show that if the damage of the internal fixation system occurs, it will first occur in the stress concentration area of screws and titanium plates, which should be paid enough attention. Furthermore, there is a stress concentration between the titanium mesh and the end plate in the ACTPS group, and the long-term high stress contact may lead to the sinking of the titanium mesh at a later stage.

## 4. Discussion

ACDF is one of the standard treatments for cervical disc disease. However, VBS used in ACDF is held by a single layer of cortical bone of vertebra mainly made of cancellous bone.^[29,30]^ ACSPS has obtained reliable clinical outcomes in patients with single-level cervical disc disease. But when it comes to multilevel cervical disc disease and ACCF, especially in elderly patients with osteoporosis, ACSPS tends to be unstable and may lead to nonfusion or implantation failure.^[31,32]^ According to the literature, the failure rate of multilevel operation can be as high as 20% to 50%, 10% to 20% of which need revision surgery.^[33,34]^ ACTPS, as an alternative to ACSPS, aimed to strengthen reconstruction stability. Although some previous studies suggested that ACTPS is a feasible, safe and effective method,^[6–9]^ there was no ACTPS finite element model for 2-level corpectomy to investigate biomechanical features of the design of the ACTPS plate-screw system.

In our study, we used the finite element analysis to show that ACTPS offers an alternative method for anterior 2-level corpectomy. We focused on the validation of the intact model at first. A 3-dimensional FE model of a C3–C7 segment was established, which consisted of detailed and accurate components of the cervical spine, including cortical bone, cancellous bone, end plates, annulus fibrosus, nucleus pulposus, posterior facets, intervertebral ligaments, and others. The intact model was validated through a comparison of ROM. The ROM of the intact model fell within the standard deviation of the published in vitro experimental and finite element analysis data.

Many factors affect the screw purchasing forces at the bone-screw interface, which depends not only on the way of fixation, but also the length and diameter of the screws, cortical thickness of purchase, and the bone mineral density (BMD). The motions at the bone-screw interface played a key role in screw loosening. BMD and the purchasing cortical thickness of screws were key factors in determining the pullout strength of the screws. In our study, both the ACTPS model and ACSPS model have stress concentrated at the joints of the screw and plate, while the maximum stress value of ACTPS model was lower than ACSPS model at axial rotation, which may be correlated to the fact that ACTPS provided stronger purchasing forces. The previous study reported similar results from in vitro experiment.^[35]^ Koller et al proved that the pull-out strength of ACTPS is 2.5 times more than that of VBS.^[9]^ Therefore, two 30-mm length ACTPS with two 16-mm lengths VBS in the ACTPS system rather than four 16-mm length VBS in ACSPS may account for the results. Although the screw anchorage may be enhanced, the probability of screw-plate fixation failure still exists.

The optimal endplate stress for graft fusion is still undefined but an increase in stress between the graft and endplate may be favorable for graft fusion. However, excessive stress for the graft or endplate could result in graft displacement and endplate fracture. Owing to the rigid stability of ACTPS, the ROM at C4–C7 decreased, which would help reduce load through the titanium mesh and endplate, leading to less fixation failure compared with the ACSPS model. Our study showed that compared with the ACSPS model, the maximum stress in the ACTPS model was lower under flexion-extension, axial loading, but not in lateral bending. These data suggest that the ACTPS may provide greater stability than the ACSPS. It may not be prone to stress concentration and fatigue cracking under flexion-extension and axial loading. Given this, the present results are important for clinical practice, especially when rigid fixation is needed through an anterior approach. Such conditions include anterior cervical revision surgery and patients with osteoporosis or ankylosing spondylitis, where multilevel corpectomy is required. As a result, ACTPS can serve as an alternative to ACSPS. Contradicting to the previous consensus that restricted motion due to fixation and fusion was compensated by the adjacent motion segment, the ROM at C3/4 in the ACTPS model decreased. However, whether it could predict the change or degeneration of the adjacent segment disc needs to be study in future studies.

The anterior pedicle screws were inserted smoothly during the simulation surgery, which can be difficult in a real surgery due to the limited exposure. Additionally, there are vital structures near the pedicle, such as the spinal cord, nerve roots and vertebral artery, which will lead to disastrous consequences if injured. These factors hinder the clinical application of ACTPS. Considering the preliminary results in the previous studies,^[6–10]^ we still choose the positive attitude. Moreover, with the implementation of intraoperative navigation, rapid prototyping technique and 3-dimensional printing,^[36,37]^ it would be easier for surgeons to master the skill and promote wider use of ACTPS in clinical practice.

Although the present study can reveal the biomechanical properties of the models, it still had some defects. In the first place, instead of including the C7/T1 disc, we fixed the inferior endplate of C7, which contradicts the normal state and might have interfered with the results. Additionally, we simplified the material properties, making bony and ligamentous structures isotropic and homogeneous, and regarded all the structures as healthy objects without degeneration. We will conduct further study in this area, taking more complicated physiological conditions into account.

## 5 . Conclusions

In summary, ACTPS and ACSPS are biomechanically favorable. In the reconstruction surgery of the subaxial cervical spine after 2-level corpectomy, the stability of ACTPS may be better and the risk of failure for internal fixator is relatively low compared with ACSPS fixation, except under lateral bending conditions. Based on finite element analysis, ACTPS may be a better solution for cases requiring multi-level anterior cervical reconstruction combined with osteoporosis and revision after anterior cervical spine surgery. In the future, more refined finite element models are needed to produce more reliable and relevant results.

## Acknowledgments

We thank the Medical and Health Technology program of Zhejiang Province of China (Grant No. 2018KY725); Ningbo Natural Science Foundation Zhejiang, China (Grant No. 202003N4279, 2019A610244, and 2019A610241); Ningbo medical key discipline (Grant No. 2022-B01); the Natural Science Foundation of Zhejiang, China (Grant No. LQ21H060002); the Ningbo Scientific Project of Public Welfare Zhejiang, China (Grant No. 2021S105).

## Author contributions

Conceptualization: Jie Li

Data curation: Yilei Chen

Formal analysis: Jinjiong Hong

Investigation: Minzhe Zheng

Methodology: Binhui Chen

Project administration: Liujun Zhao

Resources: Dikai Bei, Tengdi Fan

Software: Jie Li

Supervision: Liujun Zhao, Fengdong Zhao

Validation: Fengdong Zhao

Visualization: Kaifeng Gan

Writing–original draft: Jie Li

Writing–review & editing: Jie Li
